# Wheat blast: A review from a genetic and genomic perspective

**DOI:** 10.3389/fmicb.2022.983243

**Published:** 2022-09-08

**Authors:** Md. Motaher Hossain

**Affiliations:** Department of Plant Pathology, Bangabandhu Sheikh Mujibur Rahman Agricultural University, Gazipur, Bangladesh

**Keywords:** pathotype, genetic diversity, host resistance, genome analysis, genome editing

## Abstract

The newly emerged wheat blast fungus *Magnaporthe oryzae Triticum* (*MoT*) is a severe threat to global wheat production. The fungus is a distinct, exceptionally diverse lineage of the *M. oryzae*, causing rice blast disease. Genome-based approaches employing *MoT*-specific markers are used to detect *MoT* field isolates. Sequencing the whole genome indicates the presence of core chromosome and mini-chromosome sequences that harbor effector genes and undergo divergent evolutionary routes. Significant genetic and pathotype diversity within the fungus population gives ample potential for evolutionary change. Identifying and refining genetic markers allows for tracking genomic regions with stable blast resistance. Introgression of quantitative and R gene resistance into popular cultivars is crucial to controlling disease in areas where the pathogen population is diverse and well established. Novel approaches such as CRISPR/Cas-9 genome editing could generate resistant varieties in wheat within a short time. This chapter provides an extensive summary of the genetic and genomic aspects of the wheat blast fungus *MoT* and offers an essential resource for wheat blast research in the affected areas.

## Introduction

**FIGURE 1:**
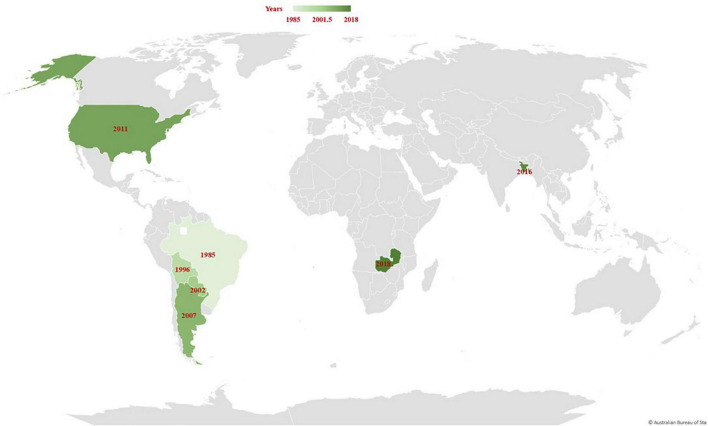
A map depicting the global appearance and spread of wheat blast over time.

Wheat blast, or “brusone,” is a relatively new fungal disease caused by the *Magnaporthe oryzae Triticum* (*MoT*) pathotype (synonym *Pyricularia oryzae*). The disease was first documented in the Paraná state of Brazil in 1985 (Igarashi et al., 1986), and it most likely emerged through a series of “host jump” from a local grass (Castroagudin et al., 2017; Inoue et al., 2017; Table 1). Following its emergence in Paraná, the wheat blast pathogen raced to neighboring states of Sao Paulo, and Mato Grosso do Sul in 1986, the Rio Grande do Sul in 1987, Minas Gerais in 1990, Goias in 1992, and Brasília in 1993 (Igarashi, 1991; Prabhu et al., 1992; Anjos et al., 1996). The disease then gradually expanded throughout South American wheat-growing regions, reaching eastern Bolivia in 1996, eastern Paraguay in 2002, and northern Argentina in 2007 (Figure 1 and Table 1). For decades, the disease has been a significant constraint on wheat productivity, particularly in the middle Cerrado region of Brazil, where the humid, subtropical climate promotes disease development. Outside of South America, wheat blast was first recorded in the United States in 2011 on a single plant in Princeton, KY, which was assumed to have arisen from an endemic *Lolium*-infecting pathogen rather than an exotic introduction from South America (Farman et al., 2017; Table 1). Later on, Bangladesh reported the first wheat blast outbreak outside South America in 2016 (Figure 1). The disease is assumed to have been introduced to Bangladesh through wheat grain imports from Brazil. While in Africa, the wheat blast was first spotted in the Zambian rainfed wheat production system in 2018 during the rainy season (Figure 1). The wheat blast was particularly prevalent in farmer-grown wheat fields and experimental plots at Malashi in the Mpika district of Muchinga Province (Tembo et al., 2020). Although the origin of the disease in Zambia is not yet known, pathogen-contaminated seeds may have contributed to its introduction in these regions.

**TABLE 1:** Emergence and spread of wheat blast in different countries.

Continent	Country	Region	Year of the first report	Mode of spread	References
South America	Brazil	Paraná	1985	Host jump from a local host	Igarashi et al., 1986
	Bolivia	Santa Cruz	1996	Introduced	Barea and Toledo, 1996
	Paraguay	Alto Parana, Itapua, Caaguazu, Caazapa, Canindeyu, and Guaira	2002	Introduced	Viedma and Morel, 2002
	Argentina	Chaco and Corrientes	2007	Introduced	Cabrera and Gutierres, 2007
North America	United States	Kentucky	2011	Host jump from *Lolium*	Farman et al., 2017
Asia	Bangladesh	Kushtia, Meherpur, Chuadanga, Pabna, Jessore, Jhenaidah, Bhola, and Barisal	2016	Introduced	Islam et al., 2016; Malaker et al., 2016
Africa	Zambia	Mpika district, Muchinga province	2017–18	Introduced	Tembo et al., 2020

**FIGURE 2:**
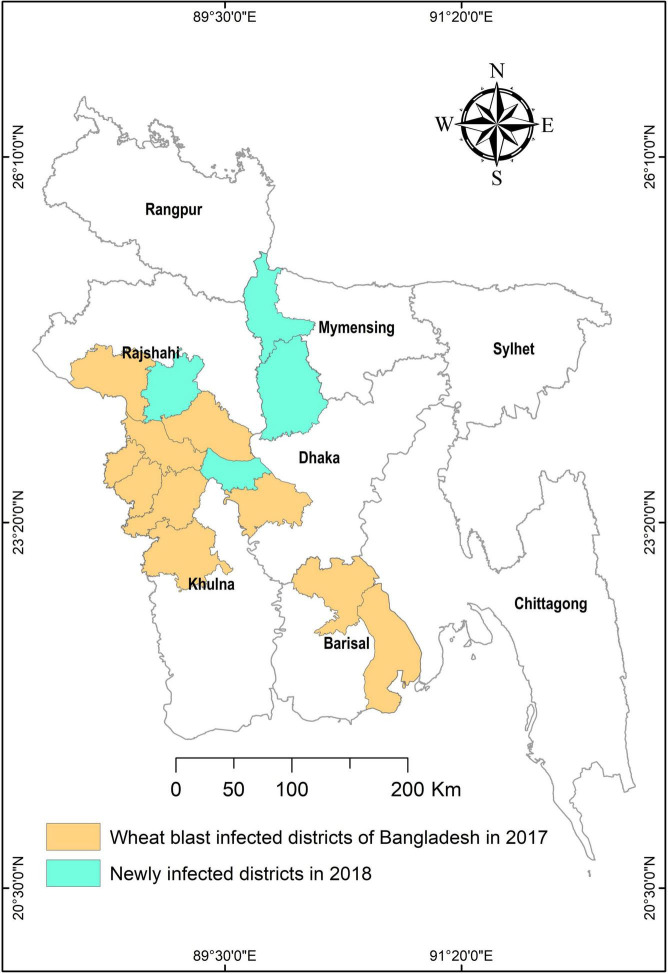
A map showing blast-infected areas in Bangladesh in 2017 and 2018. The figure is produced using the data of Yesmin et al. (2020).

The outbreak in Bangladesh is the largest ever since its first epidemic in 1985. The pandemic was extensive in the southern districts of Kushtia, Meherpur, Chuadanga, Jhenaidah, Jessore, Barisal, and Bhola (Figure 2), affecting approximately 3.5% of the total wheat area of Bangladesh (Islam et al., 2016; Malaker et al., 2016). Now, the wheat blast has established itself as a permanent problem in some of the newly infected areas and is viewed as a serious threat due to its potential for further spreading to other wheat-growing regions (Figure 2). The wheat blast is a dreadful disease that can lead to a catastrophe and cause up to 100% crop loss under favorable disease conditions. In 1997, Bolivia recorded a 69% crop loss due to a significant wheat blast epidemic (Mottaleb et al., 2018). In recent years, the wheat blast has resulted in yield losses of 10–100% in the Southern Cone region of South America (Duveiller et al., 2016). In 2016, the wheat blast outbreak in Bangladesh lowered wheat yield by 5–51% in affected areas (Islam et al., 2016). With such deadly potential, a large-scale spread of the disease might endanger world food security.

The causal organism of the wheat blast, *M. oryzae* is a haploid, filamentous, ascomycetous fungus (Couch and Kohn, 2002). The phylogenomic analysis reveals that *M. oryzae* isolates from wheat (pathotype *Triticum*), rice (pathotype *Oryza*), turfgrass (pathotype *Lolium*), finger millet (pathotype *Eleusine*), and foxtail millet (pathotype *Setaria*) are genetically distinct and form separate pathotype groups. Each pathotype exhibits a low level of pathogenicity on alternative hosts (Makaju et al., 2016; Cruz and Valent, 2017). That is why the pathotype *Triticum* does not cause disease in rice. It is believed that non-host resistance is governed by specific gene-to-gene interactions between host resistance (R) genes and race-specific avirulence (AVR) genes (Anh et al., 2015). In plant disease management programs, R-AVR gene interactions are exploited to limit disease in the field. However, the potential of the pathogen to rapidly evolve into new pathotypes or races may affect the use of R genes. Deployment of rapid and accurate pathological and molecular diagnostic tools is necessary for continuous monitoring and surveillance of the pathogen population.

Wheat blast is a complicated disease to manage, and no single strategy can achieve a satisfactory level of control. Chemical control techniques have been shown to be ineffective in controlling wheat blast when the disease pressure is high (Goulart et al., 2007; Kohli et al., 2011). In addition, resistance to strobilurin and triazole fungicides has already been detected in Brazil (Castroagudin et al., 2015; Dorigan et al., 2019). To address these concerns and achieve sustainable disease management, utilizing blast-resistant wheat cultivars is the most preferred method (Cruz and Valent, 2017). However, developing wheat cultivars resistant to blast requires the identification of dependable genetic resistance. Several studies have already evaluated blast resistance responses and identified a few blast resistance genes with varying levels of efficacy. Novel biotechnological solutions such as genetic engineering, genome editing, or gene stacking can also be used to increase the effectiveness and durability of host genetic resistance to the pathogen. Such initiatives could benefit from an in-depth understanding of the genetic and genomic aspects of wheat blasts. This review summarizes the current genetic and genomic updates on wheat blast, giving a valuable resource for wheat blast research in affected areas.

## *Magnaporthe oryzae*, the cause of the deadly blast

*Magnaporthe oryzae*, formerly known as *M. grisea* is the causal agent of the blast. The fungus is a species complex that infects over 50 grass species, including rice (*Oryza sativa* L.), wheat (*Triticum aestivum* L.), barley (*Hordeum vulgare* L.), oats (*Avena sativa* L.), perennial and annual ryegrass (*Lolium species*), finger millet (*Eleusine coracana*), Italian (foxtail) millet (*Setaria italica*), and crabgrass [*Digitaria sanguinalis* (L.) Scop]. The fungus was named *Pyricularia* after the pyriform shape of the asexual conidia of *P. grisea* on crabgrass (Saccardo, 1880). The rice isolates were later classified as *P. oryzae* (Cavara, 1892). According to the hosts of *Pyricularia* species, *P. oryzae* was designated for rice isolates, while *P. grisea* was for all other cereal and grass isolates (Sprague, 1950). The sexual form of *P. grisea* from *Digitaria* was identified in the laboratory and subsequently named *Magnaporthe grisea* based on the ascospore morphology (Barr, 1977; Couch and Kohn, 2002). Extensive analysis of the pathogenicity, mating compatibility, and RFLPs of *Pyricularia* isolates from a variety of hosts revealed that isolates from *Oryza, Setaria, Panicum, Eleusine, Triticum*, and *Lolium* form a genetically close, interfertile group (the CC crop isolate group), distinct from the crabgrass isolates originally designated *P. grisea* (Kato et al., 2000; Tosa et al., 2004). They proposed that the CC group be renamed *P. oryzae*. Couch and Kohn (2002) used a multilocus phylogenetic analysis to confirm the tight association amongst agriculturally significant CC isolates and classify these pathogens into the distinct species *M. oryzae*, while *M. grisea* was preserved for isolates pathogenic to *Digitaria* species. The 2011 decision that each fungus should have a unique name created a conundrum for blast researchers because of the widespread use of both *Pyricularia* and *Magnaporthe*. As a result, the community has agreed to keep *Magnaporthe* as an official synonym of *Pyricularia*, and both names will be used in the future (Zhang N. et al., 2016). A subset of the wheat pathogen population was recently combined with pathogens from finger millet and other grasses to form a new species, *Pyricularia graminis tritici*, separating the wheat blast population into two species (Castroagudin et al., 2016).

The asexual conidia of the fungus are pyriform in shape and range in color from hyaline to pale gray (Cruz and Valent, 2017). Each of the conidia has three cells with identical nuclei. The sexual form of the fungus is a Pyrenomycete, producing primarily four-celled ascospores in randomly arranged asci within long-necked perithecia (Cruz and Valent, 2017). Fully fertile strains are hermaphrodites that are self-sterile, with mating compatibility determined by alternate alleles of the mating-type locus *MAT1*. At a temperature of 20°C and in the presence of light, highly fertile hermaphroditic strains mate as females and males in crosses with hermaphroditic strains of the opposite mating type. Sexually viable *M. orzyae* strains also produce a Phialophora-like anamorph in which phialides are converted into small, crescent-shaped microconidia (Chuma et al., 2009). Although these microconidia germinate at low levels and infect plants via wounds, their function in nature is uncertain (Zhang et al., 2014). On hydrophobic surfaces, both conidia and ascospores germinate and generate appressoria. Appressoria that form in water droplets, such as dew, generate extremely high turgor pressure to penetrate and colonize the host leaf surface. In non-adapted hosts, fungal strains frequently failed to penetrate, and when they did, they elicited cytoplasmic granulation or hypersensitive-like reactions suggestive of gene-for-gene exchanges (Araujo et al., 2016).

## Field diagnosis and epidemics of wheat blast

**FIGURE 3:**
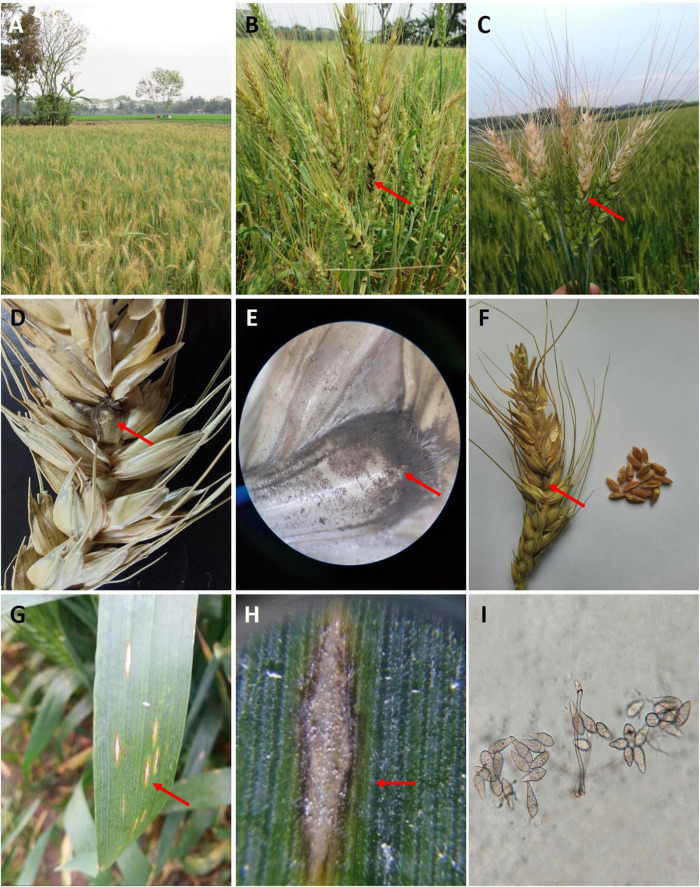
Infected wheat plants showing typical wheat blast symptoms and signs. **(A)** A severely blast infected wheat field showing silvery bleached spikes with green canopies in Meherpur District in Bangladesh. **(B)** Wheat blast symptoms on wheat heads having dark-colored infection points. **(C)** Typical partial or full bleached spikes in the field. **(D)** Dark-gray sporulation of the fungus *MoT* on the blighted rachis. **(E)** Infected glume with dark-gray sporulation of the fungus *MoT*. **(F)** Severely shriveled or wrinkled wheat grains from the blast-affected spike. **(G)** Typical elongated or elliptical lesions on wheat leaves. **(H)** A typical elliptical lesion with white to tan centers and a reddish-brown margin on a mature leaf. **(I)** Two-septate hyaline to pale gray-colored pyriform conidia of *MoT* under a compound microscope (magnification 400 ×).

Due to the prevalence of wheat blast in South America, Bangladesh, and now Zambia, there is rising concern that *MoT* strains could spread to other regions of the world. Effective surveillance of such spread will require rigorous scouting efforts and a method for quickly and precisely identifying the pathogen in suspect samples (Pieck et al., 2017). Typically, the diagnosis of wheat blast on suspect samples relies on classical disease diagnostic methods, which include visual confirmation of disease symptoms and the pyriform conidia of the fungus (Figure 3). The wheat blast fungus can infect all above-ground parts of wheat. However, the most significant infection occurs on the wheat spikes or peduncles at the reproductive stage (Figure 3A). The initial symptoms develop as a black spot or discoloration at the base or lower parts of the rachis (Figures 3B–D). An infection in the rachis or peduncle can block the transportation of nutrients to the upper spikelets above the infection points (Cruz and Valent, 2017). This eventually damages all of the top spikelets above the infection spots, causing partial or complete bleaching and drying of the spike, although the leaves may remain green (Figures 3C,D). These manifestations are considered the most distinctive symptoms of wheat blast and are used to diagnose the disease in the field. In highly susceptible cultivars, gray or dark-gray or black sporulation of the fungus can be observed at the point of infection of the rachis (Figure 3E; Islam et al., 2016). The grains produced in the blast-infected heads are small, shriveled, and deformed with a low-test weight (Figure 3F), which becomes unfit for human consumption (Malaker et al., 2016). However, infection occurring before anthesis or at an early flowering stage can cause total sterility of spikes, resulting in seed abortion (Urashima et al., 2009). On the leaves, elliptical, gray to tan necrotic lesions with dark borders frequently coexist with other foliar diseases, particularly spot blotch lesions (Figures 3G,H). Additionally, the blackening of lower nodes in the stem is noticed in certain fields. The wheat blast-infected plants display partial and complete bleached spikes with a green canopy, closely resembling Fusarium head blight (FHB) caused by *Fusarium graminearum*. That is why the wheat head blast is frequently misdiagnosed with FHB. However, FHB-infected spikes usually have pink or peach fungal spore masses, whereas blast-infected spikes do not, instead having dark-gray sporulation (Tembo et al., 2020). Moreover, the grayish mycelium of the fungus can be seen on the rachis of many spikes (Malaker et al., 2016). Incubation of blast-infected spikes/leaves in wet conditions develops 2-septate hyaline pyriform conidia (Figure 3I). Disease inspections involving simple visual detections of disease symptoms alone can be inadequate in the field because of asymptomatic colonization and since most pathogens can invade visually inaccessible tissues. Moreover, any visual assessment and human evaluation of the disease phenotypes in the field requires considerable expertise and trained personnel. Such procedures are often time-consuming and prone to human bias. Recent advances in digital technologies for detecting, diagnosing, and quantifying plant diseases may provide partial solutions to issues related to visual disease evaluation. In particular, the newly developed sensor-based technologies have enabled the early detection of plant diseases over a large area (Mahlein, 2016). However, sensor-based technologies have not yet been deployed for in-field early detection of wheat blast.

The simultaneous presence of vulnerable wheat plants and virulent *MoT* strains in the same location does not always guarantee widespread infection and the development of a wheat blast epidemic. Environmental conditions substantially impact the epidemic by influencing the host plant and the pathogen. Moisture and temperature are the two most significant environmental variables affecting the availability, growth stage, succulence, and genetic vulnerability of the host plant, and survival, vigor, multiplication, sporulation, dissemination, germination, and penetration of the pathogen. Wheat blast epidemics are favored by rainy and humid weather conditions. Severe field infections occur in seasons with continuous rainfall during anthesis, with an average temperature of 18–25°C, followed by a period of sunny, hot, and humid weather (Kohli et al., 2011). Cardoso et al. (2008) observed that an optimal temperature between 25 and 30°C and a surge in wetness over 25–40 h could result in a significant outbreak of the disease. Some blast researchers and wheat growers in Bolivia and Bangladesh have reported the presence of the first hotspots within wheat fields, which could result in a blast epidemic (Islam et al., 2020). Additionally, sporulation of *MoT* from a very low initial inoculum level before spike initiation may provide sufficient secondary inoculum, resulting in head blast epidemics (Cruz et al., 2015). Numerous reports indicated that temperature rise, particularly during the winter season (Hossain and da Silva, 2013), increases the risk of wheat blast in Bangladesh. Another disease-promoting environmental element could be substantial dewfall throughout the winter, which retains excess moisture on wheat plants for 16–17 h, promoting fungal sporulation. In South America, significant outbreaks of the wheat blast are seen in humid and warmer regions such as Bolivia, Paraguay, and northwestern Argentina (Kohli et al., 2011). Temperatures between 25 and 30°C, combined with high relative humidity and frequent leaf/spike wetness owing to continuous rain, stimulate the development of wheat blast (Cruz and Valent, 2017; Mottaleb et al., 2018). In Bangladesh, there was a significant increase in the minimum temperature in all regions in 2016 (Islam et al., 2019). This rising trend in temperature combined with rainfall during the flowering season could have aided the 2016 wheat blast outbreak in Bangladesh.

## Molecular detection of *Magnaporthe oryzae Triticum* using genetic markers

Accurate and rapid detection of plant diseases is critical for minimizing crop yield losses on qualitative and quantitative levels. Detecting and discriminating wheat infecting *MoT* from other *M. oryzae* pathotypes causing blast in various Graminaceae plants has historically been difficult due to their close morpho-pathological similarities. The conventional method of determining different pathotypes of *M. oryzae* depends on pathogenicity tests. The biological analyses of host-pathogen interactions and host range studies are crucial for species delimitation. However, fast and reliable *MoT* detection using these techniques is challenging since *MoT* often does not develop visible symptoms in wheat until the heading stage. Typically, it can be identified when the infection is already at an advanced stage. Therefore, molecular diagnosis using pathotype-specific markers and comparative genome analysis have been developed to detect wheat blast timely and accurately. Additionally, molecular approaches can be utilized to quantify the inocula present within host tissue to assess the severity of the disease, although the extent to which the inoculum develops within the host is not always proportional to the disease intensity (Nutter, 2001).

An earlier study developed a polymerase chain reaction (PCR) assay using multilocus housekeeping genes to differentiate closely related *Puccinia graminis tritici* and *MoT* (Castroagudin et al., 2016). However, housekeeping genes (e.g., *MPG1* hydrophobin) appear to be conserved. Therefore, while the *MPG1* gene has been utilized in other research to distinguish isolates with comparable morphobiometrical properties, it is unlikely to be employed as a differential marker for identifying *MoT* (Tembo et al., 2020). Pieck et al. (2017), other hand, detected DNA markers that are specifically associated with *MoT* strains for PCR assay. When tested against DNA from 284 *M. oryzae* strains from 11 host species collected from several countries, one of the markers, MoT3, demonstrated specificity with *MoT*. The marker was constructed using the WB12 sequence, a segment of the MGG_02337 gene (a short-chain retinol dehydrogenase 8) from *M. oryzae*. Yasuhara-Bell et al. (2018) adapted the MoT3 marker to a loop-mediated isothermal amplification (LAMP) assay, enabling rapid detection of *MoT* under laboratory and field circumstances. Gupta et al. (2019), on the other hand, were unable to distinguish between rice and wheat blast in Bangladesh using MoT3 primers, raising concerns regarding the widespread use of MoT3 primers to identify wheat blast. Subsequently, Yasuhara-Bell et al. (2019) published a letter to the editor of Phytopathology confirming that the MoT3 assay was capable of discriminating wheat and rice isolates from Bangladesh and throughout the world when tested at three different laboratories in the United States of America (Kansas State University, University of Kentucky, and USDA-ARS, Ft Detrick, MD). A recent analysis of the polymorphism among 81 previously assembled *Magnaporthe* genomes revealed that the MoT3 sequence is absent in all *MoT* strains (Thierry et al., 2020b). Recently, the MoT3 marker was used to demonstrate that *MoT* causes blast in a variety of additional hosts, including triticale, barley, and durum (Roy et al., 2021). However, this marker may give misleading negative results for *MoT* isolates lacking the MoT3 locus, such as BR0032. In this context, additional research should be performed to identify a marker other than MGG 02337 that can be used to distinguish closely related *M. oryzae* lineages. Recently, the long-range genome sequencing of eight isolates of *M. oryzae* from four different hosts (wheat, rice, foxtail millet, and goosegrass) was made publicly available (Peng et al., 2019; Win et al., 2019). These genomic data can significantly aid in developing an improved detection test.

To address this issue, a highly sensitive new DNA marker, C17, was identified for the *Triticum* lineage (Thierry et al., 2020b). Using the primer pair C17-forward (5′-CGATAGAAACTTGAGGAAGATCAAGTAAG-3′) and C17-R reverse (5′-TCACCGAGATGCCAC-3′) primers and the C17-P probe (5′-FAM-TCGCTAACAATGTCCACCCCGCC-BHQ1-3′), a real-time quantitative PCR (qPCR) assay was developed to detect the *MoT* lineage with high sensitivity (Thierry et al., 2020b). Subsequently, a tool kit with C17 was developed for routine PCR, quantitative PCR (qPCR), and LAMP analysis of *MoT* in seeds (Thierry et al., 2020a). The toolkit’s efficiency is promising, as it can detect 100 percent of the target at a rate of infection as low as 0.25 percent, although it produces false-positive results for certain non-*MoT* isolates (Thierry et al., 2020a,b). It is still unknown whether both MoT3 and C17 are effective in the field for rapid detection of *MoT* isolates in infected wheat plants. Kang et al. (2021) found two DNA segments, MoT-6098 and MoT-6099, that are present in the *MoT* genome but not in the genome of the rice-infecting *Magnaporthe oryzae Oryzae* (*MoO*) pathotype. They established a LAMP method to detect *MoT* under isothermal conditions without using a PCR machine. The Cas12a ssDNase activation was combined with recombinase polymerase amplification (RPA) and nucleic acid lateral flow immunoassay (NALFIA) to develop a rapid diagnostic method for detecting *MoT*-specific DNA sequences in infected wheat plants. The method is accurate, sensitive, and cost-effective. Unfortunately, the quick diagnostic tool also produced false-positive results. As a result, there is no perfect diagnostic tool for *MoT*, and it is desirable to employ many markers for cross-validation (Singh et al., 2021).

## Genetic basis of host specificity of wheat blast

The blast fungus shows high levels of host-specificity and contains several lineages. Rice is considered a non-host for *M. oryzae* strains isolated from wheat. Based on distinct DNA-fingerprinting profiles, the absence of cross-pathogenicity between wheat- and rice-derived strains, and sexual incompatibility between the two host-specialized populations, it was determined that the wheat- and rice-derived populations of *M. oryzae* were genetically distinct and host-specific (Bruno and Urashima, 2001). Although *M. oryzae* isolates obtained in nature are generally specialized for specific host species, some isolates appear to cross-infect different host species (Tosa et al., 2004). Additionally, laboratory studies indicate that some hosts, such as annual ryegrass, tall fescue, and weeping lovegrass, are “universal susceptibilities” for infection by fungal strains belonging to many pathotypes (Kato et al., 2000; Tosa et al., 2016). While barley is very vulnerable in the laboratory, there are just a few field reports of barley blast, presumably because of the cooler areas where barley is grown. Recently, *MoT* was shown for the first time to cause barley blast in Bangladesh (Roy et al., 2021).

Understanding the genetic basis for *MoT* strain host specificity is critical for disease management as alternative hosts may serve as a source of inoculum for wheat and as reservoirs for the long-term survival of the pathogen. While little is known about wheat blast disease, research on rice blast disease has revealed a large number of effector genes, which often encode tiny proteins that are produced selectively in plants and play a role in host invasion. Certain effectors, known as *AVR* effectors, play a critical role in determining host species specificity by blocking infection in response to the recognition by corresponding host species-specific R genes and inducing hypersensitive resistance (Yaegashi, 1978; Yaegashi and Asaga, 1981; Valent et al., 1991; Tosa et al., 2016). Strains of *M. oryzae* pathotypes cannot infect weeping lovegrass, *Eragrostis curvula*, because a host-specific *AVR* effector, *PWL2* in *M. oryzae*, prevents carrier strains from infecting weeping lovegrass (Sweigard et al., 1995). *AVR1-CO39* is an *AVR* gene in rice that was apparently acquired by an ancestor *M. oryzae* strain and later deleted from the *Oryza* pathotype via a transposon-mediated deletion event (Tosa et al., 2006). Almost all wheat-infecting *M. oryzae* isolates carry *AVR1-CO39*, but it was not amplified from the rice-infecting isolates (Maciel et al., 2014). These 69 wheat-infecting isolates were tested avirulent on Maratelli rice, confirming that *AVR1-CO39* confers AVR on *Oryza* spp. Due to a similar reason, all wheat-infecting isolates lack the *AVR-PITA* gene, while the gene is detected in the rice-infecting isolates (Maciel et al., 2014). Five *AVR* effector-like genes (*PWT1–5*) isolated from *Oryza, Setaria*, and *Avena* isolates independently inhibit infection of wheat (Tosa et al., 2006). Growing wheat varieties lacking the R gene *Rwt3* in Brazil probably allowed *MoL* strains with the matching host species-specific *AVR* effector *PWT3* to acclimatize to wheat, and the succeeding loss of *PWT3* function played a role in the broader emergence of the *MoT* subgroup (Inoue et al., 2017). Two gene pairs responsible for the incompatibility of a *Lolium* isolate on wheat were identified (Vy et al., 2014). The *MoL* AVR gene *A1* and its corresponding wheat R gene *Rmg6* and the AVR gene *A2* and its wheat R gene *R2* block the infection of wheat. The wheat R gene *Rmg1* prevents *Avena* isolates from infecting wheat, and two wheat R genes, *Rmg4* and *Rmg5*, prevent *Digitaria* isolates from infecting wheat independently (Anh et al., 2015).

When *M. oryzae* strains from wheat were mated with isolates from *Eleusine coracana* (Chlorideae), *Brachiaria plantaginea* (Paniceae), and *Setaria indica* (Paniceae), they were able to infect Poaceous plants from six different tribes and produce full perithecia (Urashima et al., 1993). Extensive genetic investigation of a cross between a rice pathogen and a weeping lovegrass disease identified AVR genes influencing rice cultivar specificity and minor genes regulating the lesion size on rice (Valent et al., 1991). Accordingly, loss of AVR genes is likely to result in a host jump event, allowing more success at individual infection sites, while the selection of favorable minor pathogenicity genes increases aggressiveness on the new host. The rice-infecting *MoO* population appears to have evolved due to a host jump from *Setaria* pathogens about the time rice was domesticated 7,000 years ago (Couch et al., 2005). Early analyses excluding *Lolium* isolates indicated that wheat isolates were more closely linked to finger millet pathogens than rice pathogens (Urashima et al., 1993). Diseases affecting *Lolium* ryegrass and wheat have been reported before the emergence of pandemic populations that have crossed continents. For example, the blast of *Lolium* ryegrass was first described as a novel disease in Louisiana in 1971, but it did not become a persistent problem until it was identified as GLS in Pennsylvania in 1991 (Rush and Carver, 1973). Interestingly, wheat interplanted with ryegrass in Louisiana at that time was also infected with *M. oryzae* (Rush and Carver, 1973). *M. oryzae* was later reported on wheat in India and Pakistan (Mcrae, 1922; Malik and Khan, 1943). These findings show that blast disease occurs on some hosts periodically before becoming a persistent problem.

## Population structure of the pathogen and gene flow

Understanding the genetic and pathotype diversity within a pathogen population is crucial for designing a resistant breeding strategy against the pathogen. According to a risk model analysis (McDonald and Linde, 2002), a pathogen with a high degree of genetic and pathotype diversity appears to have the most evolutionary potential and is the most difficult to manage. Until now, only a few studies have examined the genetic and pathotype diversity of wheat blast pathogen. Nonetheless, these few published research have demonstrated the existence of genetic and pathotype variability within *MoT* populations (Urashima et al., 1993). It has been suggested that two separate pathogen populations were responsible for wheat blast epidemics in 1998 in Brazil, one sexually fertile and one sterile (Urashima et al., 2005). DNA fingerprinting with MGR583 transposon sequences identified two distinct clonal lineages in the Paraná isolates, characteristic of asexual evolution (Urashima et al., 1999). In comparison, the majority of isolates from a wheat field in the Brazilian state of Mato Grosso do Sul mated as highly fertile hermaphrodites and exhibited high strain-to-strain variation without evidence for distinct clonal lineages. However, the *M. oryzae* isolates responsible for gray leaf spots in Japan formed two distinct populations, one sexually fertile and one sterile (Tosa et al., 2004). Maciel et al. (2014) used 11 microsatellite loci to elucidate the population structure of the wheat blast pathogen in wheat fields in central-western, south-eastern, and southern Brazil. Despite a relatively large clonal percentage, no subdivision was observed among the wheat-infecting populations. The three most common *M. oryzae* virulence groups were present at similar frequencies among the geographical regions (Maciel et al., 2014). Despite a relatively large clonal percentage, no subdivision was observed among the wheat-infecting populations. The two mating-type idiomorphs (MAT1-1 and MAT1-2) were observed in all communities except for So Paulo, but their frequencies were unequal. In all groups studied, the prevalence of MAT1-1 outweighed the presence of MAT1-2. Both mating types in a population show historical recombination (Couch et al., 2005), but the prevalence of a single mating type indicates episodic sexual stage development in most populations. The assays for diversity in virulence in wheat-infecting *M. oryzae* strains from Brazil demonstrate variability in virulence and differential responses of wheat cultivars to challenge by wheat blast pathogen (Maciel et al., 2014). Complete and partial resistance was observed in seven Brazilian wheat cultivars at both the seedling and head stages of development. Seedling virulence assays on these seven Brazilian wheat cultivars classified 69 wheat blast isolates into 14 pathotypes, whereas detached head virulence assays on the same wheat cultivars classified 27 of these isolates into eight pathotypes (Maciel et al., 2014). This is consistent with the wide range in disease susceptibility observed among wheat cultivars in both seedling and heading assays. *M. oryzae* is more infectious on older wheat leaves than the younger ones (Cruz et al., 2015). However, this contrasts with the rice blast pathosystem, which is virulent on immature expanding rice leaves (Ghatak et al., 2013). Therefore, wheat blast resistance screening should include both immature and mature plant stages.

Population structure can develop due to a lack of dispersal (i.e., limited gene flow due to distance) or a lack of adaptation (i.e., limited gene flow due to differences in the capacity to exploit resources), both of which are influenced by a plethora of factors. Understanding the influence of gene flow on population structure in fungal plant pathogens is a major objective of evolutionary microbiology. Significant differentiation between wheat and rice-infecting *M. oryzae* populations in Brazil indicates little gene flow between the two host populations (Maciel et al., 2014). Conversely, no subdivision observed among the wheat-infecting populations across Brazil is consistent with extensive gene flow over a large spatial scale. The widespread gene flow among Brazilian wheat fields is indicative of a mixed reproductive system of wheat blast fungus incorporating both sexual and asexual reproduction. It is believed that host-specific clones evolved through sexual recombination and are then selected to become specialized, infecting specific wheat varieties and then dispersing locally (Maciel et al., 2014). In contrast, field populations of the rice blast pathogen often constitute a small number of clonal lineages and lack signs of sexual recombination (Saleh et al., 2012). Female sterility and early postmating genetic incompatibilities are substantial barriers to gene flow between these two lineages. Although the *MoT* population in Bangladesh is clonally descended from a single strain of a single mating type (Islam et al., 2016), the sexual fertility of Bangladeshi strains and the degree of diversity within the blast population remain unknown. Moreover, it is essential to ascertain whether the Bangladeshi *MoT* strains can cross with indigenous strains on other grasses and strains from the fertile rice pathogen population found in the Himalayan foothills (Zeigler, 1998).

The high levels of gene flow across various geographically separated populations are mainly owing to two mechanisms: (i) human-mediated movement of infested seed and (ii) efficient long-distance pathogen dissemination via airborne inoculum (Maciel et al., 2014). Since wheat blast typically attacks the wheat heads, infected seeds have long been regarded as the principal source of primary inoculum and local dissemination. In naturally contaminated wheat fields, the percentage of infected seeds ranged from 68 to 83% (Urashima et al., 2009). Moreover, it has been demonstrated that *M. oryzae* conidia can spread more than 1,000 meters from an infected field site (Urashima et al., 2007). Bangladesh, Zambia, and Brazil were separated by several thousand kilometers. As a result, it might be impossible that the pathogen could have traveled this distance purely via air dispersal. Airborne ascospores produced during the sexual stage are likely to contribute to the pathogen’s local and regional spread, whereas contaminated seeds are thought to contribute significantly to the pathogen’s long-distance spread (Goulart et al., 1990).

## Genetic basis of wheat blast resistance

The most critical, cost-effective, and sustainable solution for mitigating wheat blast is the development of resistant wheat cultivars. Even though, until recently, no commercially available wheat variety was resistant to the blast fungus, many researchers have obtained positive results during the resistance assessment of wheat genotypes/lines against the blast fungus. Several cultivars, such as Milan, Caninde 1“S,” and BR8, showed high levels of resistance to wheat blast fungus (Ha et al., 2016). In another study, the wheat cultivar, Milan, was used in breeding programs to produce resistant varieties such as Paragua CIAT, Sausal CIAT, and Milan3/Atila/Cimmyt3 (Kohli et al., 2011; Marangoni et al., 2013). According to the Wheat Atlas database, certain newly released varieties in India (MACS-6478 and DBW-88) were developed based on the cultivar Milan.^1^ The Bangladesh Wheat and Maize Research Institute (BWMRI), with technical assistance from the International Maize and Wheat Improvement Center (CIMMYT), Mexico, has developed and launched a new wheat variety called “BARI Gom 33” (Hossain et al., 2019). The new wheat variety is a zinc-enriched (Zn) biofortified and resistant to wheat blast under field and laboratory conditions in Bangladesh (Jashore), Bolivia (Instituto Nacional de Innovacion Agropecuariay Forestal; INIAF), and the United States [United States Department of Agriculture-USDA-ARS (Agricultural Research Service) Laboratory, Maryland]. This is the first commercial wheat variety released as wheat blast resistant. Moreover, the new wheat is moderately resistant to *Helminthosporium* leaf blight and leaf rust diseases (BARI, 2017). It yields 5–8% more than existing wheat varieties in Bangladesh (Hossain et al., 2019). Other wheat cultivars, such as BRS201, BRS229, MGS3 Brilhante, and BR24, have also been found resistant to wheat blast (Maciel, 2011; Marangoni et al., 2013). Planting these cultivars may help to prevent wheat blast disease and increase wheat yield. However, wheat breeding programs worldwide are constrained in their ability to screen many lines for blast resistance, as phenotyping can only be performed in blast hotspot locations, and the number of lines that can be handled is limited unless their phenotyping capacity is expanded. Moreover, producing a commercial variety through classical breeding may take several years (5–10) at the very least.

Efforts have been made to comprehend the underlying genetics of blast resistance in potential sources. As with rice blast, genetic resistance to wheat blast is known to follow a gene-for-gene interaction model between host R genes and race-specific AVR genes within the pathogen, particularly at the seedling stage (Takabayashi et al., 2002). However, field resistance is also known to be quantitative (Goddard et al., 2020; He et al., 2021). Thus far, several blast-resistant *Rmg* (Resistance to *Magnaporthe grisea*) genes have been identified in wheat (Table 2). White cultures of *M. oryzae* with a *Triticum* isolate were avirulent on tetraploid wheat (*T. dicoccoides*) accession “KU109” (Tat4). The resistance of Tat4 to the white cultures is controlled by a single major gene, *RmgTd(t)*, which is considered a hidden resistance gene (Cumagun et al., 2014). Cytological analysis revealed that the moderate resistance controlled by *RmgTd(t)* was associated with a hypersensitive reaction of mesophyll cells. Zhan et al. (2008) identified the *Rmg2* (chromosome 7A) and *Rmg3* (6B) genes in the common hexaploid wheat (*Triticum aestivum*) cultivar Thatcher. These genes confer blast resistance at the seedling stage but are ineffective at high temperatures or during the heading stage. Tagle et al. (2015) identified *Rmg7* (2A) in a tetraploid wheat accession St24 (*Triticum dicoccum*, KU120) against Br48, a *Triticum* isolate of *M. oryzae*. Two other wheat accessions, St17 (*T. dicoccum*, KU112) and St25 (*T. dicoccum*, KU122), were resistant to Br48 and exhibited a disease response pattern similar to St24. Anh et al. (2015) identified *Rmg8* (2B) in the common hexaploidy wheat cultivar S-615. *Rmg7* and *Rmg8* recognize the same AVR gene AVR-*Rmg8* and provide resistance at both the seedling and heading stages. However, *Rmg7* was ineffective at higher temperatures (26°C), while *Rmg8* expression was operative at temperatures above 24 °C (Anh et al., 2018). *Rmg1* (syn. *Rwt4*) and *Rmg6* (syn. *Rwt3*) (1D) genes also confer resistance in wheat seedlings and heads (Inoue et al., 2017), but *Rmg6* is temperature-sensitive and ineffective above 25°C (Takabayashi et al., 2002). Cruz et al. (2016) recently reported that the 2NS/2AS chromosomal translocation derived from the wheat wild relative *Aegilops ventricosa* conferred resistance in wheat heads. The cultivars carrying the 2NS translocation exhibited up to a 72 percent reduction in disease symptoms than cultivars lacking 2NS. However, 2NS was less effective against highly aggressive recent blast isolates (Cruz et al., 2016) and ineffective in certain genetic backgrounds (Téllez et al., 2019), implying that the 2NS translocation-mediated resistance is background dependent and partial. Hence, 2NS translocation alone cannot guarantee adequate resistance against wheat blast.

**TABLE 2:** List of R genes identified against various strains of wheat blast fungus.

Name of *R* genes	Source wheat species	Accession/ Cultivar	Location of the genes	Wheat blast strain	Efficacy of the gene	References
*RmgTd(t)*	*Triticum dicoccum*	KU109 (Tat14)	–	White cultures of a *Triticum* isolate	Control moderate resistance	Cumagun et al., 2014
*Rmg1* (syn. *Rwt4*)	*Triticum aestivum*	Norin 4	Chromosome 1D	*Avena* isolate Br58	Confer resistance in seedlings and heads, but temperature-sensitive	Inoue et al., 2017
*Rmg2*	*Triticum aestivum*	Thatcher	Chromosome 7A	*Triticum* isolate Br48	Confer blast resistance at the seedling stage and are temperature sensitive	Zhan et al., 2008
*Rmg3*	*Triticum aestivum*	Thatcher	Chromosome 6B	*Triticum* isolate Br48	Confer blast resistance at the seedling stage and are temperature sensitive	Zhan et al., 2008
*Rmg4*	*Triticum aestivum*	*Norin 4*	Chromosome 4A	*Digitaria* isolate Dig41	Confer high resistance even at a high temperature (26°C)	Nga et al., 2009
*Rmg5*	*Triticum aestivum*	Red Egyptian	Chromosome 6D	*Digitaria* isolate Dig41	Confer high resistance even at a high temperature (26°C)	Nga et al., 2009
*Rmg6* (syn. *Rwt3*)	*Triticum aestivum*	Norin 4	Chromosome 1D	*Lolium* isolate TP2	Confer resistance in seedlings and heads, but temperature-sensitive	Inoue et al., 2017
*Rmg7*	*Triticum dicoccum*	St24 (KU120), St17 (KU112), St25 (KU122)	Chromosome 2A	*Triticum* isolate Br48	Confer resistance at the heading stage but ineffective at 26°C	Tagle et al., 2015
*Rmg8*	*Triticum aestivum*	S-615	Chromosome 2B	*Triticum* isolate Br48	Confer resistance at the heading stage and even at 26°C	Anh et al., 2018
*2NS*	*Aegilops ventricosa*	–	–	*Triticum* isolate Br48 but not B71	Confer resistance to head blast, but not foliar blast	Cruz et al., 2016
*RmgGR119*	Albanian wheat	GR119	–	*Triticum* isolate Br48	Confer high resistance to all *Triticum* isolates tested	Wang et al., 2018

Regrettably, *Rmg2, Rmg3*, and *Rmg7* have already been surpassed by more aggressive field *MoT* isolates (Cruz and Valent, 2017), emphasizing the critical need to identify additional sources of resistance to defeat the wheat blast fungus. Moreover, numerous studies have revealed opposing blast resistance responses at various stages of wheat development. For example, the 2NS translocation confers head resistance but is not known to offer foliar resistance against blast (Cruz et al., 2016). A study of 85 wheat cultivars grown in the United States of America demonstrated that resistance to blast at the seedling stage is not always a reliable indicator of resistance at the heading stage (Cruz et al., 2012). A weak negative correlation was observed between disease severity at the seedling and heading stages in Argentinian wheat cultivars (Martinez et al., 2019). While these studies demonstrate varietal differences in seedling and head infection resistance, little is known about the genetic basis of these differences. Therefore, a better understanding of the genetics underlying blast resistance is critical to ensuring the persistence of resistance throughout wheat development.

Since the majority of *Rmg* genes were ineffective against *MoT* when tested at temperatures over 26°C, or against recently collected aggressive strains, or at the critical head stage, the search for a new resistance gene or gene combination continues. During a screening of a global collection of 520 local landraces of common wheat, a highly resistant accession, GR119, was discovered in an Albanian collection (Wang et al., 2018). GR119 had two resistance genes that conferred resistance to the wheat blast fungus additively; one was *Rmg8*, and the other was a novel gene tentatively called *RmgGR119*. However, the degree of control is still not optimum in Bolivia. When the aggressive Bolivian *MoT* isolate 008 was used, the susceptible checks attained disease severity of 90% or more, *Rmg8* averaged 95.1%, and the combination of the two genes averaged 73.1% (Valent et al., 2021). Additional *MoT* isolates should be tested in subsequent experiments for the efficacy of these two genes. The effect of the rice R gene *Piz-t* on leaf and head blast in wheat strain T-25 was studied (Navia-Urrutia, 2020). Although two transformed wheat lines showed a considerable reduction in the percentage of leaf area impacted, none of the lines showed an increase in the head blast. Nonetheless, these findings are significant in light of the increased leaf symptoms reported in commercial fields during blast epidemic years. Additionally, despite the limited link between leaf and head blast resistance in some cultivars, new field trials indicate that inoculum from basal leaves early in the season may contribute to head infection (Cruppe, 2019). As a result, R genes that are effective during the leaf stage should be introduced into cultivars resistant to head blast.

A recent report by Juliana et al. (2022) provides results of the first-ever study to test genomic selection in breeding for resistance to wheat blast. In this study, researchers have evaluated genomic selection by combining genotypic data with extensive and precise field data on wheat blast responses for three sets of genetically diverse wheat lines and varieties, totaling more than 700, grown over several crop cycles at locations in Bangladesh and Bolivia. The study has also compared predictions made using thousands of genome-wide markers with those made using a small number of molecular markers connected to the 2NS translocation. The results show that genotyping utilizing one-to-few markers tagging the 2NS translocation is sufficient to predict the blast response of wheat lines in an environment where the translocation governs wheat blast resistance. They have also found that selection based on a small number of wheat blast-associated molecular markers preserved 89% of lines that were also selected using field performance data and eliminated 92% of those that were eliminated based on field performance data. Thus, marker-assisted and genomic selection provide viable alternatives to the slower and more expensive field screening of numerous thousands of wheat lines in disease hotspot locations and can hasten the development of blast-resistant wheat varieties, particularly in the early stages of breeding. The findings can aid in predicting which wheat lines are likely to be successful in providing blast resistance for upcoming crosses and those that can be advanced to the following generation after selection.

## Wheat blast genomic analyses

Genomic information contributes to understanding the molecular mechanisms that lead to fungal pathogenicity and developing novel disease control techniques. Identifying genetic differences in fungal infections like *M. oryzae* may indicate whether the fungus can circumvent disease-resistant cultivars. Researchers around the globe are digging into wheat blast pathogen with a new *de novo* fungal genome assembly, population sequence data, and other approaches. To date, the genome of more than 50 *M. oryzae* isolates has been sequenced and is publicly available.^2^ ^,3^ Different isolates possess similar genomic sizes and overall genomic structures. The ∼40 megabase pair (Mb) genome of *M. oryzae* is transposon-rich and has around 13,000 genes spread across seven chromosomes (Zhang H. F. et al., 2016). Many genes involved in the growth and infection phase of *M. oryzae* were discovered in distinct isolates. Some strains include hundreds of isolate-specific genes and several isolate-specific duplication events; moreover, each genome contains a substantial number of poorly conserved transposon-like elements (Xue et al., 2012). Gladieux et al. (2018) used whole-genome sequence data from 76 *M. oryzae* isolates collected from 12 grass and cereal genera, including wheat and rice, to predict the genetic characteristics of *M. oryzae* lineages and to review the species status of the wheat-infecting populations. Species recognition utilizing a genealogical concordance, published data, or the extraction of previously used loci from genomic assemblies did not support the categorization of wheat blast isolates as a new species (*Pyricularia graminis-tritici*). Multiple divergent lineages within *M. oryzae* were identified, each preferentially linked with a single host genus, implying incipient speciation in response to host shift or range expansion. Gene flow analyses demonstrated that genetic exchanges contributed to the formation of numerous lineages within *M. oryzae*, even when incomplete lineage sorting was considered. The findings of this study gave a better understanding of the eco-evolutionary mechanisms underlying *M. oryzae* diversification and demonstrated the utility of genomic data for epidemiological surveillance. Peng et al. (2019) presented a nearly complete reference genome sequence of *MoT* B71, an aggressive Bolivian field isolate. The genome was assembled using Pacific Biosciences long reads and Illumina short reads. Along with seven core chromosomes found in the fungal genome, the fungal sequences fell into a dispensable mini-chromosome that contained repetitive sequences and effector gene sequences nabbed from the ends of the fungal chromosome. Together with re-sequencing data for eight more fungal isolates, their results hint that the mini-chromosome contributes to the evolution of the wheat blast pathogen’s effector repertoire. No mini-chromosome was found in an early field strain, but at least two from another isolate contain distinct effector genes and core chromosome end sequences. The mini-chromosome is densely packed with transposons, most typically seen at the ends of core chromosomes. Additionally, transposons in mini-chromosomes lack the distinctive signature of genome defenses against repeat-induced point (RIP) mutations. These findings collectively show that dispensable mini-chromosomes and core chromosomes follow distinct evolutionary paths and that mini-chromosomes and core chromosome ends are connected in the wheat pathogen genome as a mobile, rapidly changing effector compartment.

## Enhanced blast resistance by clustered regularly interspaced short palindromic repeats/CRISPR associated 9-targeted mutagenesis

Editing plant genomes to improve plant traits has always been a challenge. In recent years, it has been demonstrated that sequence-specific nucleases (SSNs), such as zinc-finger nucleases (ZFNs), transcription activator-like effector nucleases (TALENs), and clustered regularly interspaced short palindromic repeats (CRISPR)/CRISPR-associated (Cas) 9 (CRISPR/Cas9), are practical tools for crop improvement via gene-specific genome editing (Baltes and Voytas, 2015). CRISPR/Cas9 is the most effective SSN to date and has been used to modify the genomes of important crops like rice (Endo et al., 2015), maize (Feng et al., 2016), wheat (Wang et al., 2014), sorghum (Jiang et al., 2013), tomato (Ito et al., 2015), soybean (Du et al., 2016), and potato (Wang et al., 2015). The SSN-induced gene-specific DNA double-strand breaks (DSBs) are repaired mainly either by the high-fidelity homologous recombination (HR) or the error-prone non-homologous end joining (NHEJ) pathways (Symington and Gautier, 2011). NHEJ frequently introduces minor insertion or deletion (InDel) changes at the cut site, resulting in gene loss. In comparison to RNAi, SSN-based genome editing enables complete knockdown without using foreign DNA. Several successful ZFN- and TALEN-based trait improvements in key crops have been described (Clasen et al., 2016). The CRISPR/Cas9 technology is relatively simple and more cost-efficient than other approaches. The CRISPR/Cas9 system is a versatile tool for genome editing because it allows for the simultaneous targeting of numerous genes using short RNAs as guides (Doudna and Charpentier, 2014).

With the advancement in the CRISPR-Cas9 technique, it is now possible to produce customized resistance genes against wheat blast pathogen. A noteworthy example is a disruption of the rice *OsERF922* blast susceptibility gene through CRISPR-Cas9, which increased resistance to rice blast (Wang et al., 2016). CRISPR-Cas9 has been utilized to disrupt various genes in wheat, including *TaDREB2* and *TaERF3* (Kim et al., 2018), suggesting its high potential for modifying wheat blast susceptibility genes once found. Another study used CRISPR/Cas9 to alter the endogenous wheat genes *eIF(iso)4E-2* and *eIF4G*, which encode translation initiation factors that are responsible for enhanced wheat streak mosaic virus (WSMV) replication in the host (Navia-Urrutia, 2020). The targeted genes’ expression levels were decreased in edited lines compared to the control Bobwhite wild-type. However, characteristic WSMV symptoms developed in both edited-lines and Bobwhite wild-type plants, and no variations in viral accumulation were observed, implying that knocking these genes out did not affect virus infection. Hence selecting a unique target site is a valuable strategy for improving the specificity of CRISPR-based genome editing methods. The availability of wheat genomic resources and an understanding of the molecular biology underlying blast resistance response may aid in identifying target genes for wheat genome editing to confer *MoT* resistance.

## Conclusion

Wheat blast is a new severe fungal disease of wheat identified in South America, South Asia, and Africa. In favorable climatic conditions, the disease has proven challenging to control, frequently leading to catastrophic yield and quality losses. In locations where the disease is prevalent, a vital disease management strategy should entail timing the wheat planting date so that the heading does not coincide with warm rainy weather. A rapid and effective wheat blast diagnostic tool needs to be deployed to overcome the challenge of timely pathogen detection. The wheat blast forecasting model can be used to focus surveys on locations and times with favorable environmental conditions for blast disease. Critical research priorities should include continuing epidemiological studies and optimizing different management strategies. Breeding a resistant cultivar is crucial for the long-term management of the disease. Genome-wide association studies (GWAS) are essential to finding additional resistance genes as most R genes and wheat varieties are ineffective against the most recent *MoT* isolates. However, the only currently effective resistance, included in the *2NvS* translocation from the wild wheat-related *Aegilops ventricosa*, gives variable resistance levels depending on the genetic background of wheat varieties. Research efforts are urgent to identify additional resistance genes that can be stacked with *2NvS* resistance to confer broad and persistent resistance. The introduction of modern biotechnology in agriculture, either by wheat mutagenesis utilizing CRISPR/Cas9 genome editing or through genetic engineering of non-host resistant genes, may potentially prevent the pathogen’s expansion in South Asia. Updating and strengthening plant quarantine and biosecurity acts is currently the most important priority to avoid spreading the pathogen to disease-free countries through seed trade. Outreach efforts, extension activities, and education initiatives should prioritize training farmers, plant disease diagnosticians, extension workers, and research specialists in addressing global food security challenges by the disease.

## Author contributions

The author confirms being the sole contributor of this work and has approved it for publication.

## Footnotes

1
http://wheatatlas.org/varieties/

2
http://www.ncbi.nlm.nih.gov/assembly/?term=magnaporthe

3
http://genome.Jouy.inra.fr/gemo
